# Decoupled Spectral Tuning and Polarization Locking in Twisted α‐MoO_3_ Polaritonic Crystals

**DOI:** 10.1002/nap2.70299

**Published:** 2026-09-22

**Authors:** Zhichen Zhao, Jiayi Yuan, Yanzhen Yin, Gang Zhong, Junbo Xu, Qingdong Ou, Tao Jiang

**Affiliations:** ^1^ MOE Key Laboratory of Advanced Micro‐Structured Materials Shanghai Frontiers Science Center of Digital Optics Institute of Precision Optical Engineering, and School of Physics Science and Engineering Tongji University Shanghai China; ^2^ Sino‐Luso Joint Laboratory for Optoelectronics Macao Institute of Materials Science and Engineering (MIMSE) Macau University of Science and Technology Taipa Macao China; ^3^ Macau University of Science and Technology Zhuhai MUST Science and Technology Research Institute Zhuhai China; ^4^ Shanghai Institute of Intelligent Science and Technology Tongji University Shanghai China

**Keywords:** anisotropic nanophotonics, polaritonic crystals, polarization locking, twisted bilayer α‐MoO3

## Abstract

Polaritonic crystals (PoCs), formed by integrating periodic lattices with two‐dimensional polaritonic materials, provide a powerful platform for nanoscale light manipulation and far‐field spectral engineering. However, far‐field spectral engineering in existing PoCs is primarily achieved through geometric redesign, requiring structural reconfiguration to access different spectral responses. Here, we overcome this limitation by constructing a twisted bilayer α‐${\text{MoO}}_{3}$ PoC consisting of a patterned and a pristine layer, where interlayer twist enables spectral tuning without geometric modifications. Meanwhile, the principal crystal axis of the patterned layer deterministically governs the axes of polarization‐selective resonances arising from lattice‐assisted polariton excitation. This polarization‐locking behavior remains robust against interlayer twisting, enabling independent control over the spectral response and the polarization direction. These findings provide a deterministic strategy for constructing deep‐subwavelength PoCs for multidimensional infrared light manipulation, offering new opportunities for advanced flat optics and reconfigurable nanophotonic devices.

## Graphical abstract

We introduce a deterministic design strategy employing twisted bilayer α‐${\text{MoO}}_{3}$ polaritonic crystals to decouple infrared spectral and polarization degrees of freedom. Although adjusting the interlayer twist angle tunes the absorption spectra, the maximum absorption axis remains robustly locked to the patterned layer's crystal axis for precise polarization manipulation, advancing multifunctional infrared flat optics and on‐chip nanophotonic devices.

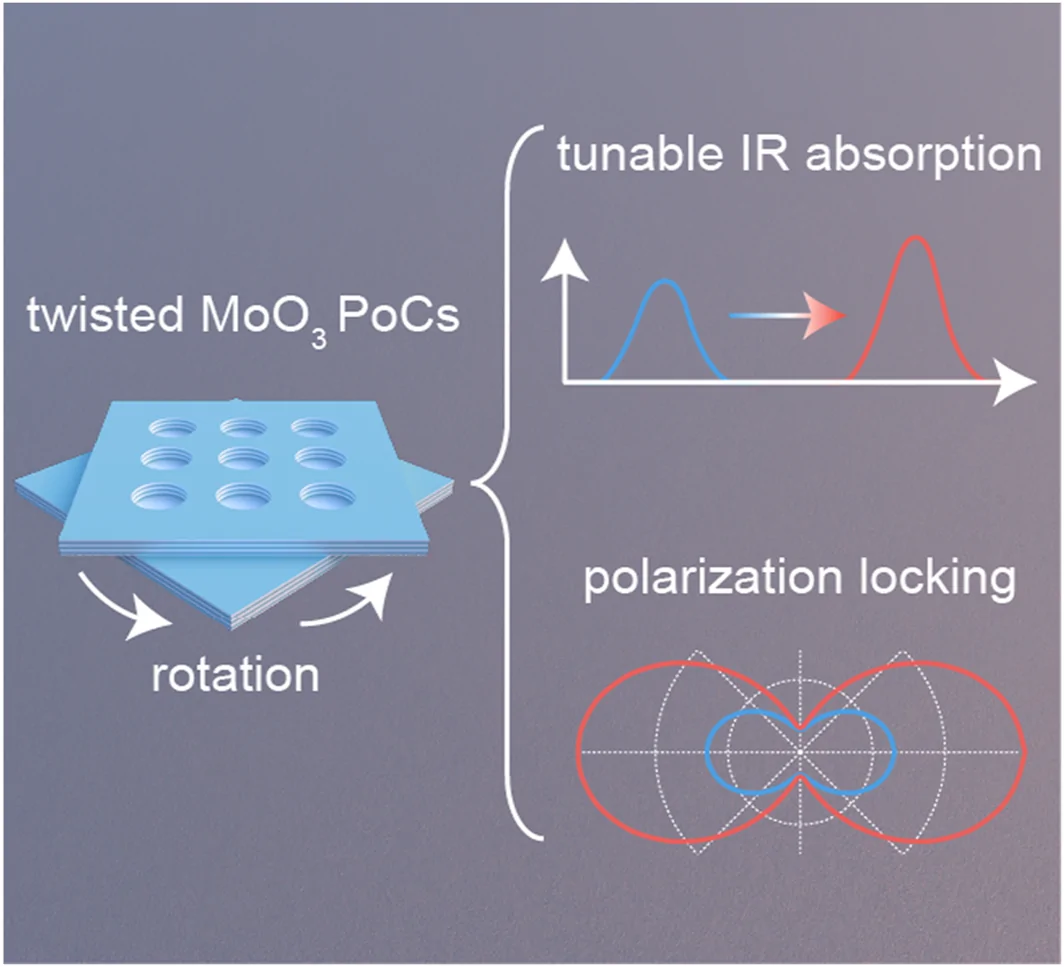

## 1 Introduction

Polaritonic crystals (PoCs), which combine periodic lattices with two‐dimensional polaritonic materials, have emerged as a powerful platform for manipulating light at nanoscale and engineering far‐field optical responses [1, 2, 3, 4, 5, 6, 7, 8, 9, 10, 11, 12, 13, 14]. By providing reciprocal lattice momentum for free‐space photons, PoCs overcome the momentum mismatch with highly confined polaritons, enabling their efficient far‐field excitation and detection [1, 9, 10, 11, 12, 13, 15, 16, 17, 18]. This capability enables free‐space launching and far‐field probing of polaritons, establishing PoCs as a versatile platform for developing advanced far‐field polaritonic functionalities. Recently, vdW materials, such as graphene [19, 20, 21, 22], hBN [9, 23, 24, 25, 26, 27], and α‐${\text{MoO}}_{3}$ [10, 11, 12, 13, 28, 29, 30, 31, 32, 33], have been widely employed in PoCs for polariton engineering. Among them, α‐${\text{MoO}}_{3}$ has attracted particular interest due to its inherent low‐loss in‐plane anisotropic phonon polaritons [34, 35, 36, 37]. Such anisotropic polaritons exhibit directional propagation and engineerable dispersion, providing diverse opportunities for nanoscale light manipulation [38, 39, 40, 41, 42, 43, 44]. The highly tunable dispersion and subwavelength light confinement of α‐${\text{MoO}}_{3}$ PoCs hold great promise for controlling spectral responses at a deep‐subwavelength scale. This capability offers a route beyond the conventional reliance on geometric scaling in traditional photonic crystals [45], which inherently limits their device miniaturization. However, far‐field spectral engineering in existing PoCs is still mainly achieved through geometric redesign, requiring structural reconfiguration to access different spectral responses. This dependence not only limits spectral tunability but also reduces the adaptability of PoC devices to different operating conditions. Therefore, overcoming these limitations is highly desirable for programmable and multifunctional polaritonic platforms.

Here, we introduce interlayer twist [46, 47, 48, 49, 50, 51, 52, 53] as an additional degree of freedom for spectral engineering by designing a twisted bilayer PoC with a patterned α‐${\text{MoO}}_{3}$ layer stacked on an unpatterned pristine layer. Driven by momentum compensation from the periodic hole array, lattice‐assisted excitation of anisotropic polaritons in α‐${\text{MoO}}_{3}$ produces pronounced polarization‐selective resonances, whose spectral response can be tailored through interlayer twist. Notably, the principal crystal axis of the patterned layer deterministically defines the polarization axes of these resonances, with the resulting polarization‐locking behavior remaining robust throughout the twist‐induced spectral evolution. Consequently, this approach enables independent control over the spectral response and polarization direction within a deep‐subwavelength PoC without geometric alterations. Our results establish a deterministic strategy for vdW PoCs capable of multidimensional infrared light manipulation, opening new opportunities for advanced infrared flat optics and reconfigurable nanophotonic devices [1, 54, 55].

## 2 Results and Discussion

In this work, we theoretically investigate the unique polaritonic mode resonances of a twisted bilayer α‐${\text{MoO}}_{3}$ PoC (Figure 1a). The system comprises a top α‐${\text{MoO}}_{3}$ layer patterned with a square lattice of holes and a pristine bottom α‐${\text{MoO}}_{3}$ layer, both with a thickness of 100nm. This heterostructure is supported by a Si substrate with a 285nm thick ${\text{SiO}}_{2}$ oxide layer. The square hole lattice with a period of 500nm and a hole radius of 100nm is oriented along the x and y axes. To describe the twisted configuration, we define $\theta_{t}$ and $\theta_{b}$ as the angles between the [001] crystal axes of the top and bottom α‐${\text{MoO}}_{3}$ layers and the y‐axis, respectively. Based on this twisted bilayer PoC, we demonstrate independent control over the spectral and polarization responses: the infrared absorption spectra can be effectively tuned by rotating the α‐${\text{MoO}}_{3}$ crystal orientations, whereas the polarization of the resonance peaks remains locked to the crystal axis that supports polariton propagation in the patterned layer (Figure 1b).

**FIGURE 1:**
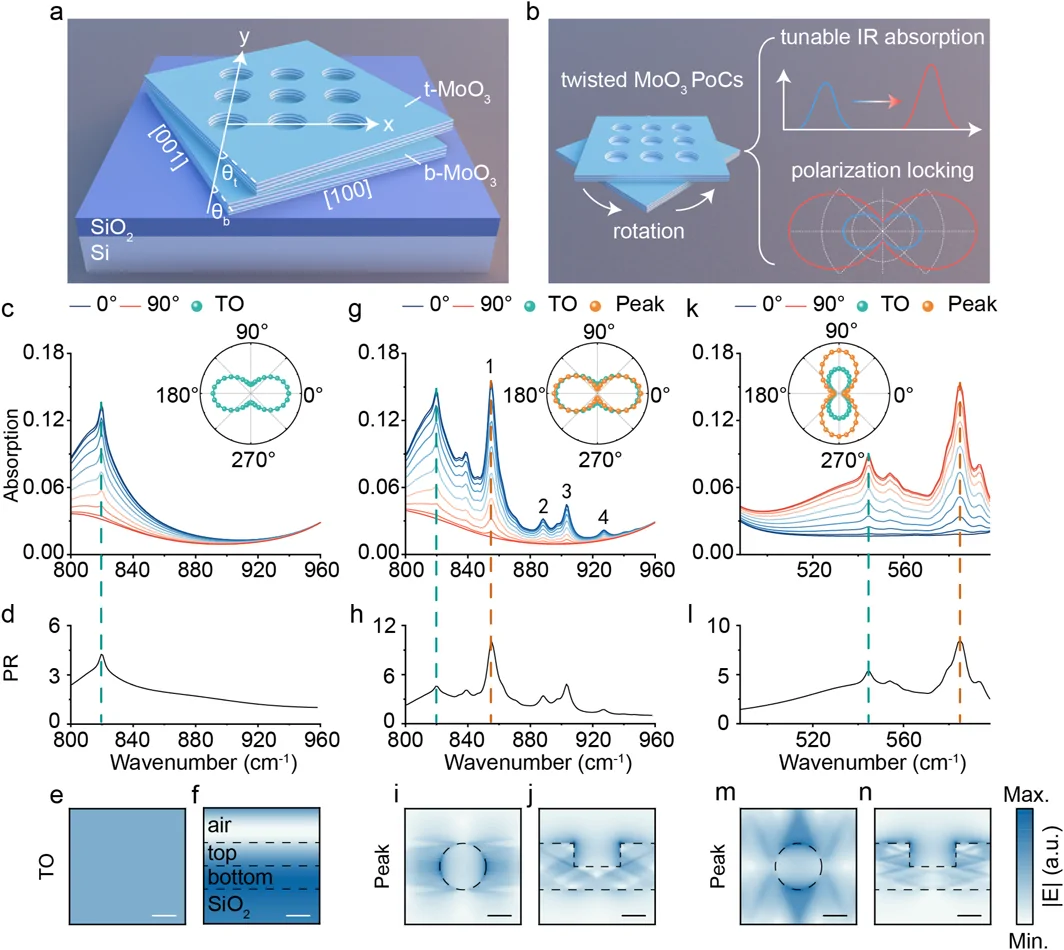
Lattice‐induced anisotropic absorption resonances in α‐${\text{MoO}}_{3}$ PoC. (a) Schematic of the twisted bilayer α‐${\text{MoO}}_{3}$ polaritonic crystal. The top layer is patterned with a square hole lattice aligned with the x and y axes. $\theta_{t}$ and $\theta_{b}$ represent the twist angles of the [001] crystal axes of the top (t‐${\text{MoO}}_{3}$) and bottom (b‐${\text{MoO}}_{3}$) layers relative to the y‐axis, respectively. (b) Diagram showing the twist‐tunability and polarization‐locking mechanism. (c) Absorption spectra of the pristine bilayer α‐${\text{MoO}}_{3}$ within the RB2 at an aligned configuration of $\theta_{t}=\theta_{b}=0{}^{\circ}$. The 0° and 90° curves correspond to excitation polarization parallel to the x and y axes, respectively. The teal dashed line indicates the TO phonon frequency. The inset shows the polar plot of the absorption at the TO phonon frequency. (d) Polarization ratio spectrum derived from the spectra in panel (c). (e, f) Simulated electric field distribution (|E|) above the pristine bilayer surface (e) and the vertical cross‐section along the x‐axis (f) under 0° excitation polarization. (g) Absorption spectra of the α‐${\text{MoO}}_{3}$ PoC in RB2 at an aligned configuration of $\theta_{t}=\theta_{b}=0{}^{\circ}$. The hole lattice introduces multiple resonances (labeled 1–4). The teal and orange dashed lines indicate the frequencies of the TO phonon peak and the primary resonance peak, respectively. The inset shows the polar plots of the TO phonon peak and the primary polariton‐induced resonance. (h) Polarization ratio spectrum derived from the spectra in panel (g). (i, j) Simulated electric field distribution above the PoC unit cell (i) and the vertical cross‐section along the x‐axis (j) in RB2 under 0° excitation polarization. (k) Absorption spectra of the identical PoC configuration in the RB1. (l) Polarization ratio spectrum derived from the spectra in panel (k). (m, n) Simulated electric field distribution above the PoC unit cell (m) and the vertical cross‐section along the y‐axis (n) in RB1 under 90° excitation polarization. Scale bars: 100nm.

We first investigate the infrared absorption resonances introduced by the periodic square lattice. For the pristine bilayer structure ($\theta_{t}=\theta_{b}=0{}^{\circ}$ without lattice), the spectral response is characteristic of a standard α‐${\text{MoO}}_{3}$ flake, showing an absorption peak at the transverse optical (TO) phonon frequency as illustrated in Figure 1c for the second Reststrahlen band (RB2) [35]. Due to the intrinsic anisotropy of α‐${\text{MoO}}_{3}$ (see Supporting Information S1: Note S1), this TO absorption peak exhibits a polarization‐sensitive response, where the absorption intensity varies with the excitation polarization. To quantitatively evaluate this optical anisotropy, we introduce the polarization ratio (PR), which is defined as follows:

(1) $$PR=\frac{I_{\max}}{I_{\min}}$$

where $I_{\max}$ and $I_{\min}$ denote the respective maximum and minimum absorption intensities obtained by varying the excitation polarization angle. The corresponding PR spectrum calculated across the investigated frequency range is presented in Figure 1d. Near‐field simulations at the TO frequency reveal the spatial electric field distributions (see Methods), showing no sub‐wavelength confinement in the absence of a momentum‐matching structure (Figure 1e,f).

The introduction of the square hole lattice into the top layer of the pristine bilayer $\left(\theta_{t}=\theta_{b}=0{}^{\circ}\right)$ significantly modifies the spectral response, giving rise to multiple resonance peaks within the frequency range supporting polariton propagation along the [100] crystal axis (Figure 1g). The lattice‐induced resonances also exhibit pronounced anisotropic features (Figure 1g and Supporting Information S1: Figure S2). Notably, the PR of the primary resonance peak is significantly higher than that of the TO peak (Figure 1h), indicating enhanced polarization selectivity. The same behavior is observed in the inverted configuration, where the bottom layer is patterned whereas the top layer remains pristine (see Supporting Information S1: Figure S3), confirming that the spectral response and polarization characteristics are robust against layer exchange. Simulated electric field distributions within the PoC unit cell at these resonance frequencies confirm the effective excitation of polaritons, suggesting that the absorption enhancement is driven by lattice‐assisted polariton launching (Figure 1i,j). To further validate this mechanism, we extended our investigation to the first Reststrahlen band (RB1), where the polariton propagation direction rotates to the [001] crystal axis [35] (Figure 1k–n). We observe that the polarization axes of both the TO and the polaritonic resonance peaks undergo a corresponding 90° rotation compared to their orientations in RB2. This orthogonal shift further highlights the strong correlation between the absorption characteristics and polariton excitation, with their polarization directions locked to the crystal axes that support polariton propagation. Furthermore, we examined the spectral response in the RB3. Due to the in‐plane hyperbolic‐to‐elliptic topological transition of polariton dispersion in this frequency regime, the optical anisotropy is largely suppressed, resulting in a near‐isotropic spectral response where the PR remains close to 1 (see Supporting Information S1: Figure S4).

To gain deeper insight into the correlation between the observed absorption resonances and the excitation of polaritons, we analyze the electric field distributions in both real and momentum space. Figure 2a–d display the simulated near‐field maps at the frequencies corresponding to the four resonance peaks identified in Figure 1g. These maps exhibit highly confined near‐field profiles characteristic of the hyperbolic phonon polaritons in α‐${\text{MoO}}_{3}$. By applying fast Fourier transform (FFT) to these near‐field images, we extract the corresponding momentum‐space distributions (Figure 2e–h). Taking Peak 1 (Figure 2e) as a representative case, the polariton momentum distribution manifests as a discrete grid of high‐intensity spots indexed by the diffraction orders $\left(n_{1},n_{2}\right)$, which correspond to the reciprocal lattice vectors provided by the square hole array [11]. Overlaid on the FFT maps are the isofrequency contours (IFCs) calculated via the extended plane wave expansion method for the bilayer α‐${\text{MoO}}_{3}$ system (see Supporting Information S1: Note S2). At the frequency of Peak 1, the polariton IFCs precisely intersect with the (±1,±2) reciprocal lattice points. This indicates that the lattice provides the necessary momentum compensation to satisfy the matching condition between free‐space photons and polaritons, thereby inducing a polaritonic Bragg resonance. As the excitation frequency increases (Figure 2f–h), the IFCs expand and evolve in shape. However, at each spectral resonance, the IFCs consistently intersect with specific reciprocal lattice points. This one‐to‐one correspondence between the spectral maxima and the momentum‐space intersections provides strong evidence that the absorption resonances originate from the lattice‐assisted excitation of polaritonic Bragg modes within the PoC.

**FIGURE 2:**
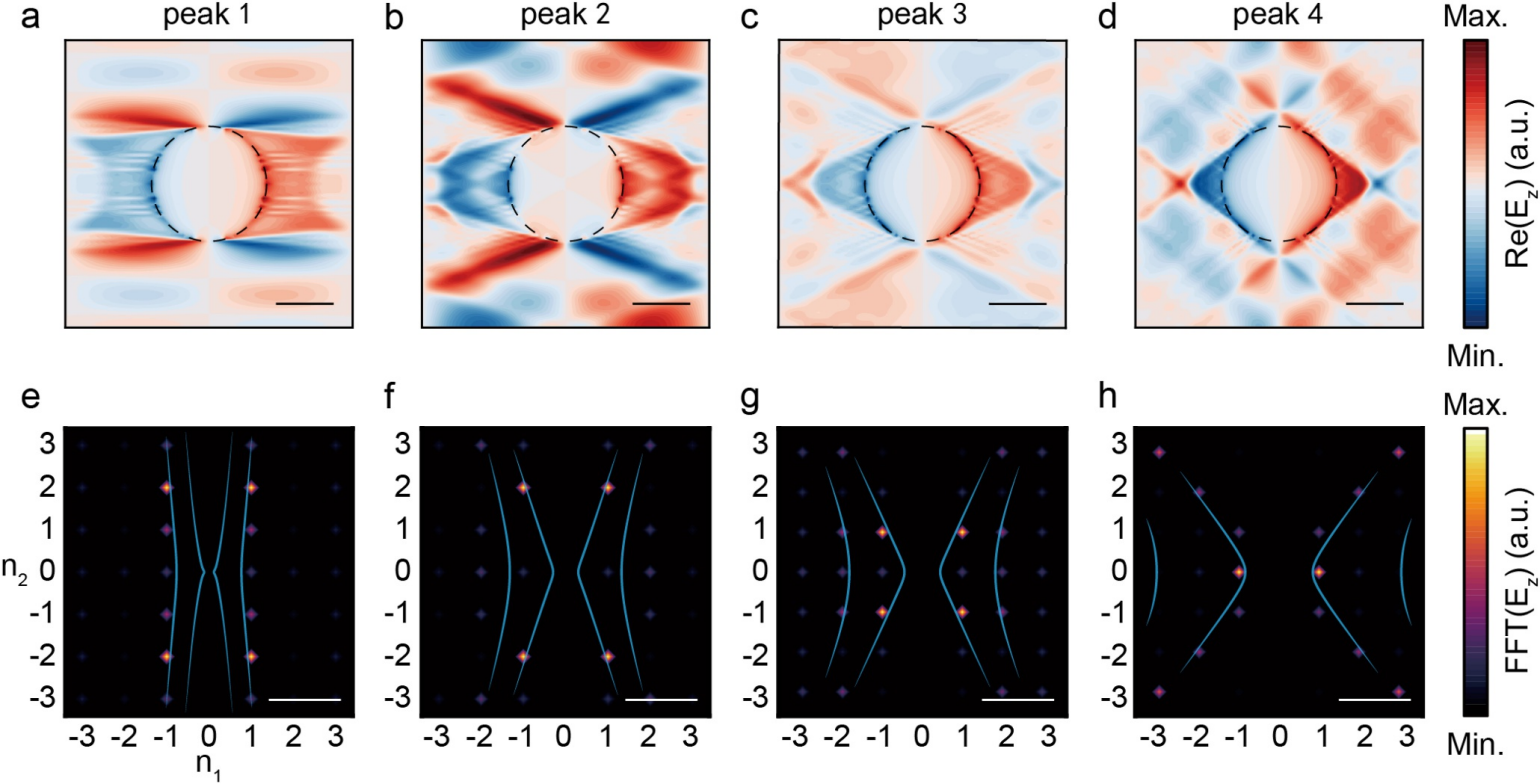
Polaritonic Bragg resonances in the α‐${\text{MoO}}_{3}$ PoC. (a–d) Simulated real‐space electric field distributions $\left(Re\left(E_{z}\right)\right)$ above the PoC unit cell. The frequencies correspond to the four resonance peaks (labeled 1–4) identified in Figure 1g. Scale bars: 100nm. (e–h) FFT amplitude maps derived from the near‐field images in panels (a–d). The cyan curves represent the calculated IFCs of the α‐${\text{MoO}}_{3}$ polaritons. Scale bars: $40k_{0}$, where $k_{0}$ is the free‐space wavevector at each respective resonance frequency.

Consequently, by varying the $\theta_{b}$ in the twisted bilayer α‐${\text{MoO}}_{3}$ heterostructure while maintaining $\theta_{t}$ fixed at 0°, we can actively tune the spectral behavior of the PoC by modulating the alignment between the polariton IFCs and the reciprocal lattice. As the bottom layer is rotated from 0° to 90°, the spectral distribution and intensity of the absorption peaks undergo a distinct evolution (Figure 3a–c). To evaluate the polarization response, we extract the polar plots for the intrinsic TO phonon peak and the primary resonance peak at their respective frequencies (Figure 3d–f). We observe a clear divergence in behavior, where the orientation of the TO phonon peak rotates significantly following the rotation of the bottom layer, whereas the polaritonic resonance peak remains locked along the [100] crystal axis of the patterned top layer. At the specific frequencies corresponding to these polaritonic resonance peaks, the momentum‐space distributions (Figure 3g–i) reveal that although altering the twist angle induces a topological transition of the bilayer polariton IFCs, which modifies their contours and orientations in momentum space, different orders of the reciprocal lattice points consistently provide adaptive matching to give rise to these different resonance absorption peaks. Along with the twist‐induced evolution of the resonance frequency, both the PR and linewidth also vary with the interlayer twist angle (see Supporting Information S1: Figure S5). The PR generally decreases, whereas the linewidth exhibits an overall increasing trend as the bilayer IFCs evolve from strongly anisotropic hyperbolic contours toward elliptical contours.

**FIGURE 3:**
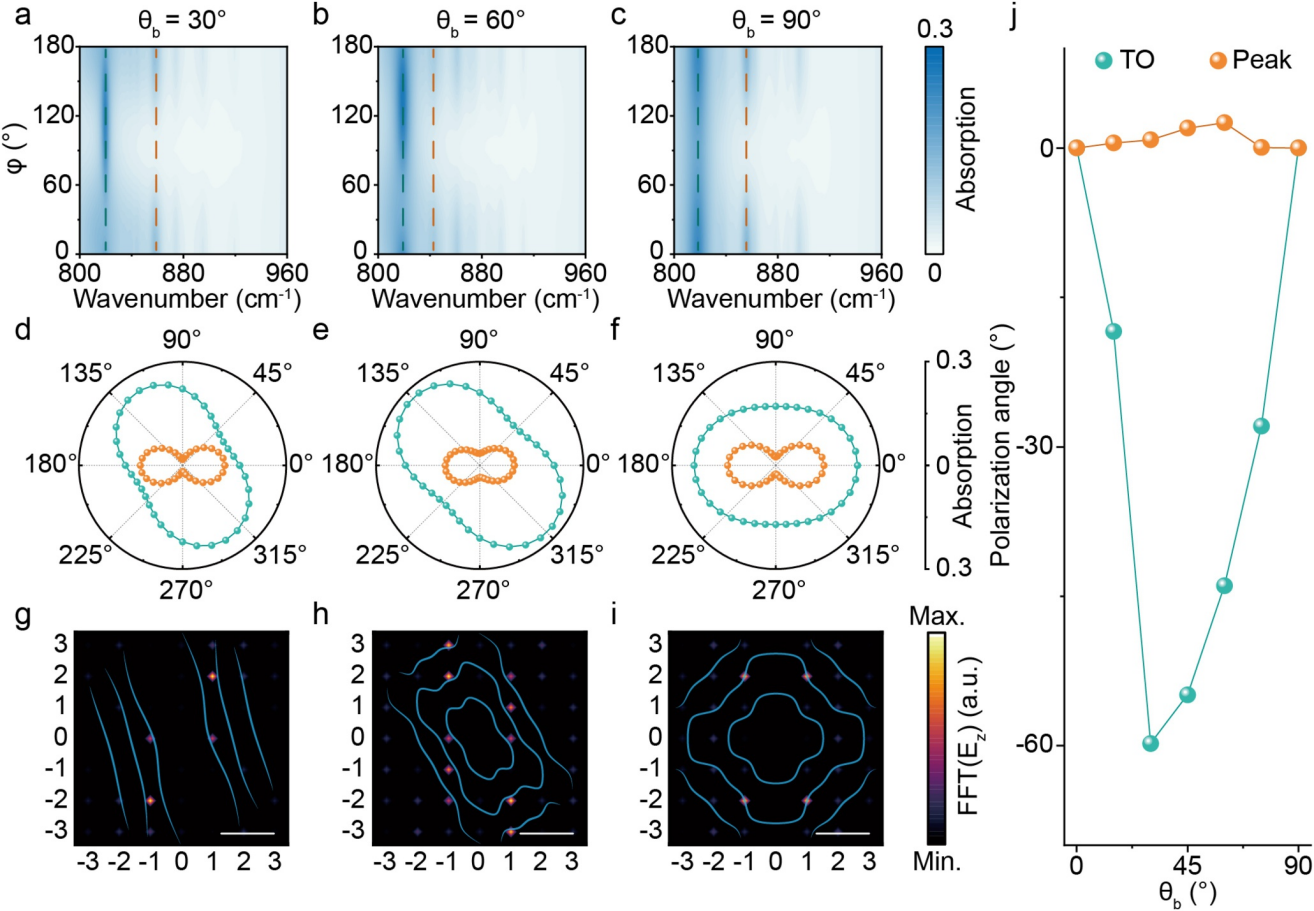
Spectral tuning of polaritonic resonances via twist‐angle modulation. (a–c) Simulated 2D contour maps of infrared absorption as a function of wavenumber and excitation polarization angle φ at bottom‐layer twist angles of $\theta_{b}=30{}^{\circ}$, 60°, and 90°. The teal and orange dashed lines indicate the frequencies of the TO phonon peak and the primary polaritonic resonance peak, respectively. (d–f) Polar plots of the absorption intensity as a function of excitation polarization angle for the TO phonon peak (teal) and the resonance peak (orange) at $\theta_{b}=30{}^{\circ}$, 60°, and 90°. (g–i) Momentum‐space FFT amplitude maps of the near‐field distributions under 0° excitation polarization at the resonance frequency for $\theta_{b}=30{}^{\circ}$, 60°, and 90°. The cyan curves represent the calculated polariton IFCs of the twisted bilayer heterostructure. Scale bars: $40k_{0}$. (j) Polarization angles of the maximum absorption axes for the TO phonon peak (teal) and the resonance peak (orange) as a function of the bottom‐layer twist angle $\theta_{b}$.

To compare the sensitivity of the intrinsic TO phonon and the polaritonic resonance responses to structural twisting, we plot the polarization angles of their maximum absorption axes as a function of $\theta_{b}$ (Figure 3j). The polarization angle is defined relative to the x‐axis, with counterclockwise and clockwise directions designated as positive and negative, respectively. This quantitative comparison highlights the excellent robustness of the lattice‐induced response, which maintains a strict polarization locking near 0° with a maximum angular deviation of only approximately 3°, despite the acute angular sensitivity exhibited by the intrinsic TO phonon peak. Additionally, this polarization locking holds with comparable precision for the other weaker resonance peaks, whose absorption polarization axes similarly maintain angular deviations within approximately 3° (see Supporting Information S1: Figure S8). This layer‐determined polarization locking can be understood from the dominant absorption contribution of the patterned layer. The periodic hole array enables efficient lattice‐assisted excitation and propagation of polaritons, resulting in substantially stronger electric field enhancement in the patterned layer than in the unpatterned layer as shown in Figure 1j,n. Since the local absorption is governed by both the material loss and the electric field intensity, the enhanced polaritons in the patterned layer dominate the overall absorption response and consequently determine the polarization axis of the absorption resonances.

To gain a comprehensive understanding and achieve effective control over the polarization locking behavior within the twisted bilayer α‐${\text{MoO}}_{3}$ PoC, we introduce the rotation angle of the patterned top layer $\left(\theta_{t}\right)$ alongside the unpatterned bottom layer $\left(\theta_{b}\right)$. Following an analysis procedure similar to that established in Figure 3, we investigate two representative top‐layer orientations, specifically $\theta_{t}=30{}^{\circ}$ (Figure 4a–f) and $\theta_{t}=60{}^{\circ}$ (Figure 4g–l), while continuously tuning the bottom‐layer orientation. The extracted polar plots and corresponding momentum‐space distributions reveal that the polarization locking direction directly follows the patterned top layer, pinning precisely at the designated $\theta_{t}$ angles. Furthermore, when the patterned structure is instead fabricated on the bottom layer, the polarization locking direction switches to track the bottom‐layer twist angle $\theta_{b}$ (see Supporting Information S1: Figure S9). This direct dependence demonstrates that the polarization locking direction in this heterostructure is entirely determined by the crystal axis direction of the patterned layer, rather than that of the unpatterned layer. Although the rotation of the unpatterned bottom layer successfully modulates the polaritonic IFC geometries to shift the resonance absorption frequencies, it leaves the polarization locking direction essentially unaffected. To verify the robustness of this polarization locking against variations in the unpatterned layer orientation, we plot the polarization angles of the maximum absorption axes as a function of the twist angle $\theta_{b}-\theta_{t}$ (Figure 4m). For both the $\theta_{t}=30{}^{\circ}$ and $\theta_{t}=60{}^{\circ}$ cases, the disturbance induced by the bottom‐layer rotation is restricted within a narrow window of ±2°, which confirms an exceptional stability.

**FIGURE 4:**
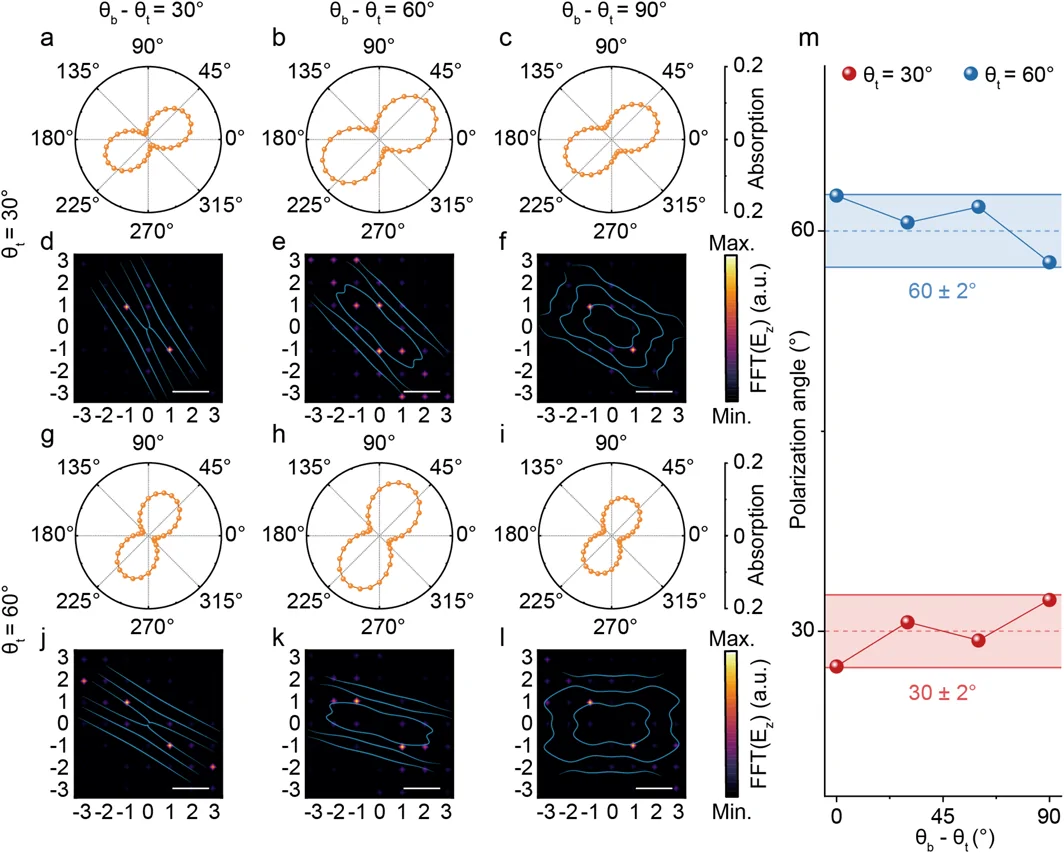
Layer‐dominated polarization locking under arbitrary dual‐layer configurations. (a–c) Polar plots of the absorption intensity as a function of excitation polarization angle for the primary resonance peak at a fixed top‐layer twist angle of $\theta_{t}=30{}^{\circ}$ with relative twist angles of $\theta_{b}-\theta_{t}=30{}^{\circ}$, 60°, and 90°. (d–f) Momentum‐space FFT amplitude maps of the near‐field distributions under 30° excitation polarization at the resonance frequencies corresponding to the configurations in panels (a–c), where cyan curves represent the calculated polariton IFCs. (g–i) Polar plots of the absorption intensity as a function of excitation polarization angle for the primary resonance peak at a fixed top‐layer twist angle of $\theta_{t}=60{}^{\circ}$ with relative twist angles of $\theta_{b}-\theta_{t}=30{}^{\circ}$, 60°, and 90°. (j–l) Momentum‐space FFT amplitude maps of the near‐field distributions under 60° excitation polarization at the resonance frequencies corresponding to the configurations in panels (g–i), where cyan curves represent the calculated polariton IFCs. (m) Polarization angles of the maximum absorption axes for the resonance peak as a function of the twist angle $\theta_{b}-\theta_{t}$ at fixed top‐layer orientations of $\theta_{t}=30{}^{\circ}$ (red dots) and 60° (blue dots), where the shaded bands represent the reference ranges of 30±2° and 60±2°, respectively.

In addition, we examined the sensitivity of the polarization locking and spectral tuning range to variations in key structural parameters, including the hole radius, lattice period, and patterned‐layer thickness (see Supporting Information S1: Figure S10). Within a fabrication deviation of ± 10% from the designed values, the deviation of the polarization angle of the primary resonance peak remains within approximately 1°. Meanwhile, the spectral tuning range remains essentially unchanged for parameter deviations within ± 5%. Overall, moderate variations in the PoC geometry have a minor impact on both the polarization locking and spectral tunability, suggesting that fabrication variations within approximately ± 5% can preserve these functionalities and highlighting the tolerance of the proposed PoC design for practical implementation.

These collective findings demonstrate an effective decoupling between spectral engineering and polarization control within the twisted bilayer α‐${\text{MoO}}_{3}$ PoC. Thus, the operating wavelength of the PoC can be independently tuned via the unpatterned layer rotation, whereas its polarization response remains exclusively fixed to the patterned layer orientation $\theta_{t}$, offering a versatile platform for multidimensional infrared light manipulation.

## 3 Conclusion

In conclusion, we have comprehensively investigated the far‐field spectral responses and polarization characteristics of a twisted bilayer α‐${\text{MoO}}_{3}$ vdW PoC. We demonstrate that lattice‐induced Bragg resonances exhibit a robust polarization locking effect, where the polarization direction remains locked to the principal crystal axis of the patterned layer. By rotating the unpatterned layer, the configuration enables flexible tuning of the spectral features via modulating the alignment between polariton IFCs and different orders of reciprocal lattice points, while leaving the polarization direction essentially unaffected. Consequently, this deep‐subwavelength scale PoC platform achieves an effective decoupling between spectral engineering and polarization control without geometric redesign. These capabilities provide a deterministic strategy for multidimensional infrared light manipulation, opening new opportunities for advanced infrared flat optics and reconfigurable nanophotonic devices.

## 4 Methods

Numerical absorption spectra and near‐field electric field distributions of the twisted bilayer α‐${\text{MoO}}_{3}$ PoCs were simulated using the rigorous coupled‐wave analysis (RCWA) and finite‐difference time‐domain (FDTD) methods within the commercial software package Ansys Lumerical. The simulation region was configured with a single unit cell of the square hole lattice aligned along the Cartesian x‐ and y‐axes. The anisotropic dielectric permittivity of α‐${\text{MoO}}_{3}$ was modeled using the Lorentz oscillator model, with detailed parameters provided in Supporting Information S1: Note S1. The refractive index of the background medium was set to 1. In the FDTD simulations, periodic boundary conditions were applied along both the x‐ and y‐directions to replicate an infinite periodic array, whereas perfectly matched layers were utilized along the electromagnetic wave propagation direction z‐axis. The total simulation time was set to 10,000 fs to ensure sufficient temporal evolution and convergence of the electromagnetic fields. A uniform mesh size of 5 nm was applied along the x‐, y‐, and z‐directions. Optical excitation was modeled using a linearly polarized plane wave source located within the air environment, propagating downward toward the PoC architecture at a defined polarization angle. The resulting near‐field profiles were recorded by a monitor positioned 5 nm above the surface of the PoC. The IFCs of the twisted bilayer α‐${\text{MoO}}_{3}$ configurations were theoretically mapped using the extended plane wave expansion method [52, 56]. This analytical framework incorporated the complete multilayered stack, comprising the twisted bilayer α‐${\text{MoO}}_{3}$ films, the ${\text{SiO}}_{2}$ oxide layer, and the Si substrate (see Supporting Information S1: Note S2).

## Author Contributions

**Zhichen Zhao:** investigation, visualization, writing – original draft. **Jiayi Yuan:** writing – original draft. **Yanzhen Yin:** writing – review and editing. **Gang Zhong:** writing – review and editing. **Junbo Xu:** methodology, writing – original draft. **Qingdong Ou:** writing – review and editing, supervision. **Tao Jiang:** writing – review and editing, conceptualization, supervision.

## Conflicts of Interest

The authors declare no conflicts of interest.

## Supporting information


Supporting Information S1

## Data Availability

The data that support the findings of this study are available from the corresponding author upon reasonable request.
