# Supplementary material for: Decoupled Spectral Tuning and Polarization Locking in Twisted α‐MoO3 Polaritonic Crystals
Source: Nanophotonics. 2026 Sep 22;15(18):e70299. doi: 10.1002/nap2.70299 (PMC13672175; doi:10.1002/nap2.70299)
Supplement: Supplementary file 1 [file NAP2-15-e70299-s001.pdf]

Supporting Information for Decoupled Spectral Tuning and Polarization Locking in Twisted α-MoO_3 Polaritonic Crystals

Zhichen Zhao,[†] Jiayi Yuan,[†] Yanzhen Yin,[†] Gang Zhong,[‡] Junbo Xu,[*,†] Qingdong Ou,[*,‡,¶] and Tao Jiang[*,†,§]

†*MOE Key Laboratory of Advanced Micro-Structured Materials, Shanghai Frontiers Science Center of Digital Optics, Institute of Precision Optical Engineering, and School of Physics Science and Engineering, Tongji University, Shanghai 200092, China*

‡*Macao Institute of Materials Science and Engineering (MIMSE), Sino-Luso Joint Laboratory for Optoelectronics, Macau University of Science and Technology, Taipa, Macao, 999078, China*

¶*Macau University of Science and Technology Zhuhai MUST Science and Technology Research Institute, Zhuhai 519031, China*

§*Shanghai Institute of Intelligent Science and Technology, Tongji University, Shanghai 200092, China*

E-mail: junboxu@tongji.edu.cn; qdou@must.edu.mo; tjiang@tongji.edu.cn

Contents

Section 1. Anisotropic optical response of of α-MoO_3

Section 2. Calculation of polariton isofrequency contours

Figure S1-11

References [1-3]

Note S1. Anisotropic optical response of of α-MoO_3

Naturally anisotropic α-MoO_3 possesses a dielectric permittivity tensor whose principal components can be quantitatively evaluated via a Lorentz oscillator model. Following the parameterizations reported in previous literature,[1] the frequency-dependent permittivity components $\varepsilon_j(\omega)$ along the principal axes are expressed as follows:

$$\varepsilon_j(\omega) = \varepsilon_j^{\infty}\left(1 + \frac{\omega_j^{\mathrm{LO}^2} - \omega_j^{\mathrm{TO}^2}}{\omega_j^{\mathrm{TO}^2} - \omega^2 - i\omega\Gamma_j}\right),\ j = x,\ y,\ z \tag{S1}$$

where ω represents the frequency of the incident light, and ε_j^{∞} denotes the high-frequency dielectric constant along the j-th axis. The parameters ω_j^{LO} and ω_j^{TO} correspond to the longitudinal and transverse optical phonon frequencies respectively, while Γ_j accounts for the associated damping rate. Here, the x, y, and z are aligned with the [100], [010], and [001] principal crystal directions of the α-MoO_3. The calculated real and imaginary components of these dielectric functions across the spectral range of interest are illustrated in Figure S1.

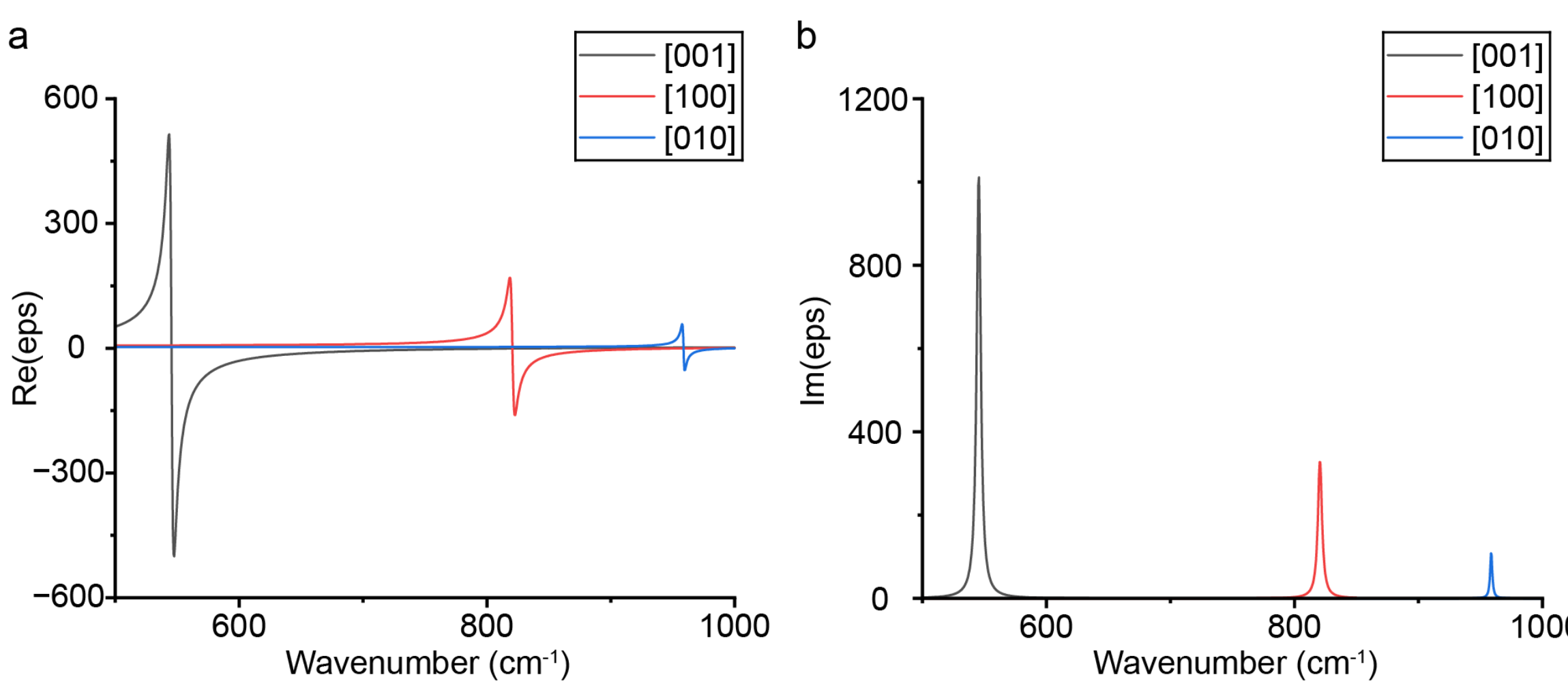

Figure S1. Real and imaginary parts of the dielectric permittivities of α-MoO_3 along the three principal crystal orientations of [100], [001], and [010].

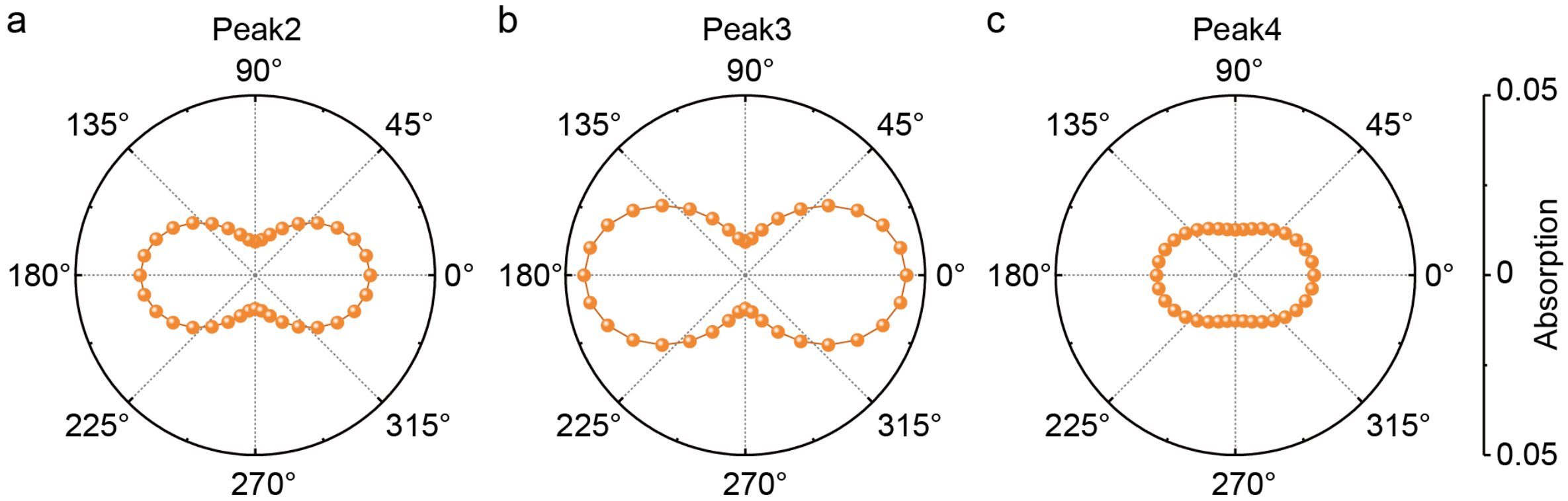

Figure S2. Supplementary absorption polar plots corresponding to resonant peak 2 (a), peak 3 (b), and peak 4 (c) identified in Figure 1g.

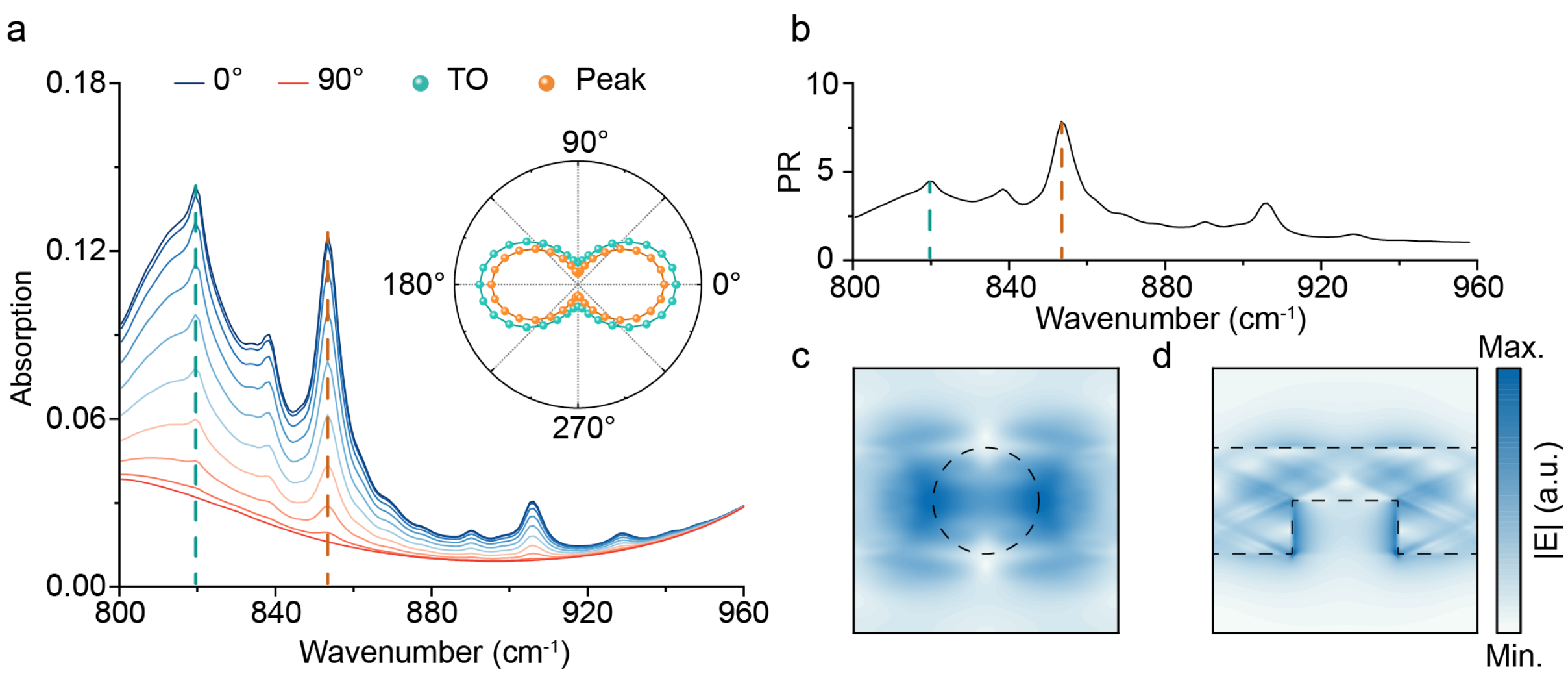

Figure S3. Far-field spectral responses and electric field distributions of the inverted bilayer α-MoO_3 configuration. The inverted configuration comprises an unpatterned top layer α-MoO_3 and a patterned bottom layer α-MoO_3. (a) Simulated polarization-dependent absorption spectra of the bilayer heterostructure featuring an unpatterned top layer and a patterned bottom layer. The teal and orange dashed lines track the TO phonon and the polaritonic resonance peak respectively, with the inset showing their corresponding absorption polar plots. (b) Polarization ratio spectrum derived from the spectra in (a). (c, d) Simulated electric field distribution above the PoC unit cell (c) and the vertical cross-section along the x-axis (d) in RB2 under 0° excitation polarization.

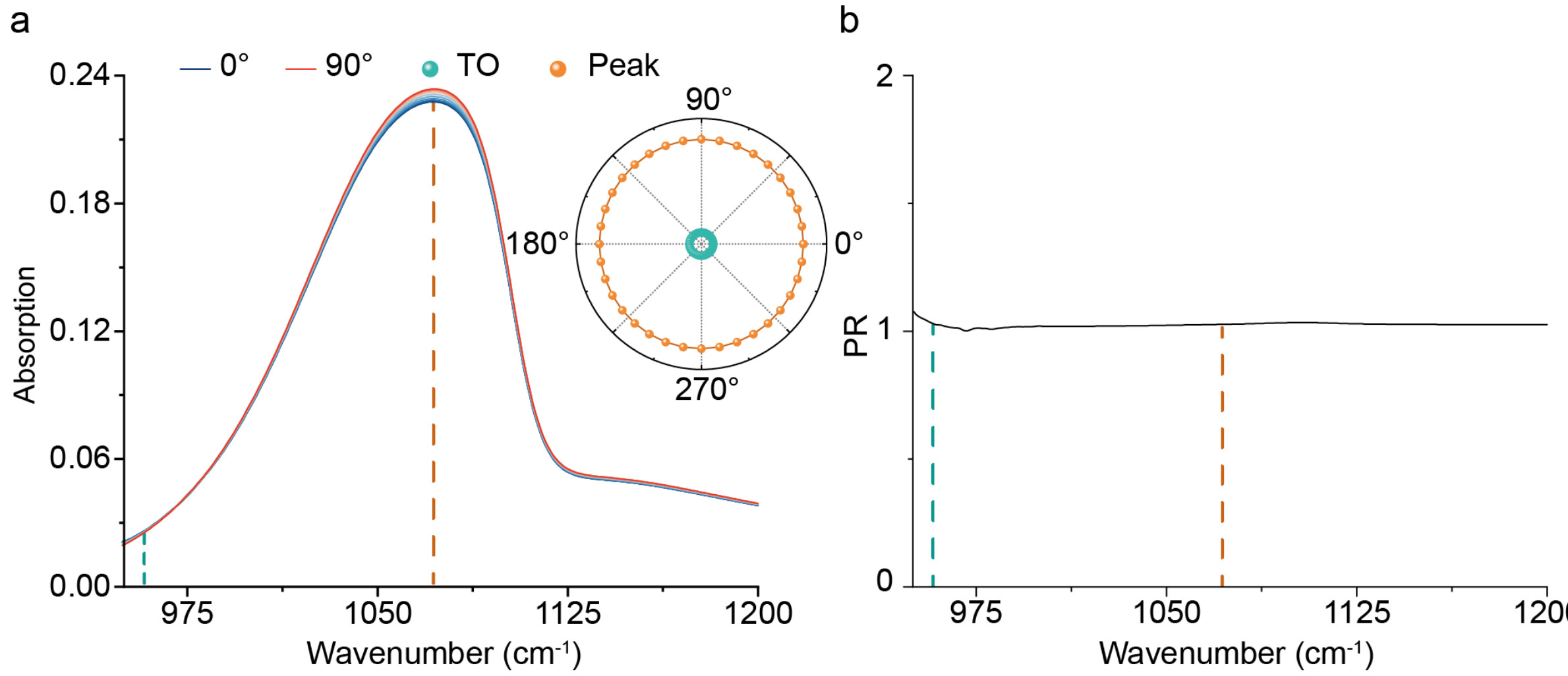

Figure S4. Far-field absorption and polarization ratio spectra of the α-MoO_3 PoC in the RB3. (a) Simulated polarization-dependent absorption spectra of the α-MoO_3 PoC in RB3. The teal and orange dashed lines track the TO phonon and the polaritonic resonance peak respectively, with the inset showing their corresponding absorption polar plots. (b) Polarization ratio spectrum derived from the spectra in (a).

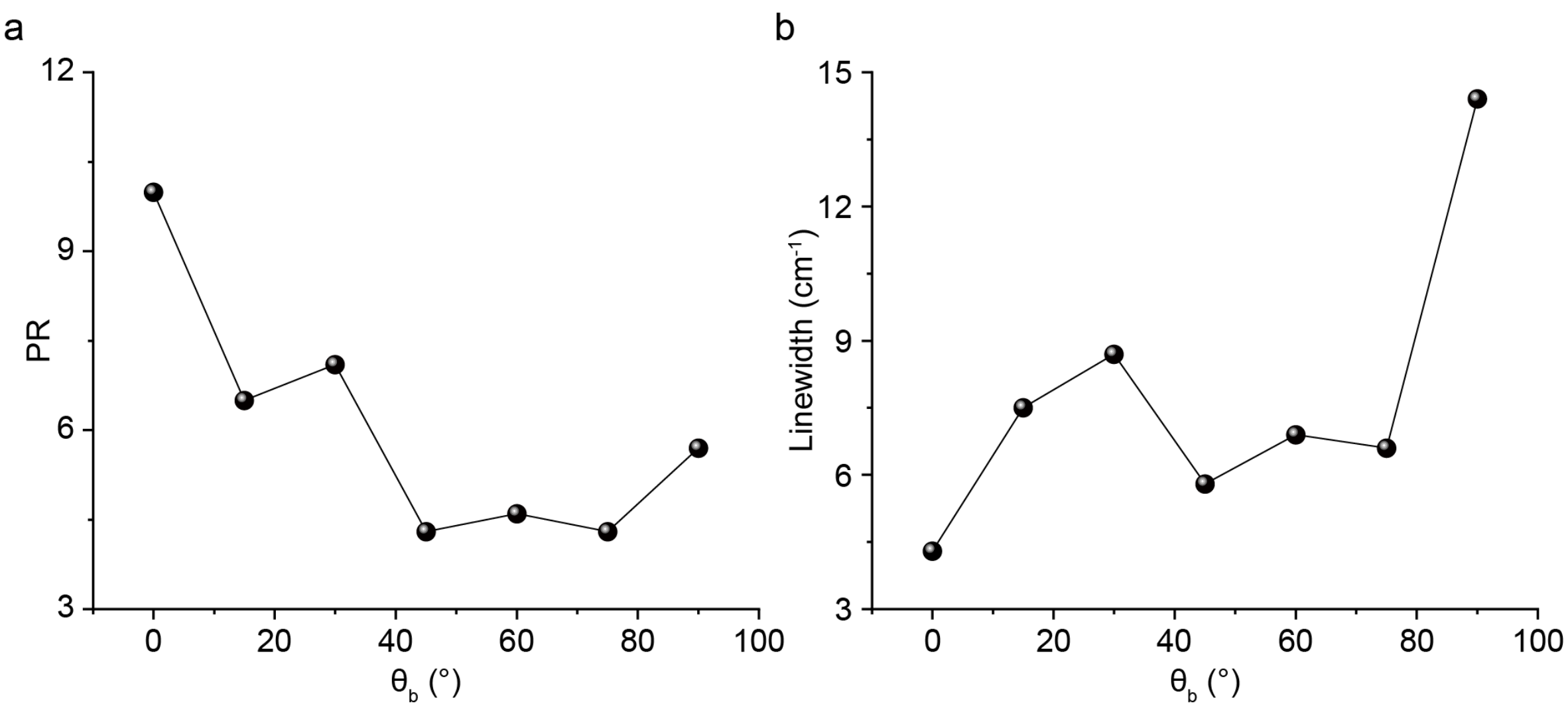

Figure S5. Polarization ratio and linewidth of the primary resonance peak as a function of the bottom-layer twist angle θ_b. The top layer is oriented at $\theta_t = 0°$.

Note S2. Calculation of polariton isofrequency contours

We employ the extended plane-wave expansion method to streamline the computation of the isofrequency contours (IFCs) of the twisted bilayer α-MoO_3 configurations.[2,3] In our study, the twisted bilayer α-MoO_3 is represented by a multilayered structure extending infinitely along the $x - y$ plane.

To adapt the EPWE method to our framework, we treat the multilayered structure as a fundamental unit and replicate it along the z direction to introduce an artificial periodicity a_z (Figure S4 a). Due to the highly localized nature of the HShP modes along the z direction, inter-unit coupling effects can be disregarded as long as the periodicity is sufficiently large (e.g., $a_z = 3000$ nm). This negates the requirement for additional Perfectly Matched Layers (PML) at the boundaries.

We establish a primary Cartesian coordinate system xyz, aligning the [100], [001], and [010] crystallographic orientations of the bottom α-MoO_3 layer to the x, y, and z axes, respectively (Figure S4b). The angle between the [100] crystallographic orientations of the top and bottom layers is defined as the twist angle θ. Furthermore, a local Cartesian coordinate system kez is established in momentum space, where the k axis represents the direction of the in-plane wavevector. The angle between the x axis and the k axis is denoted as φ.

Initially, we normalize the magnetic field intensity H within the structure unit, assuming a non-magnetic medium where $\mu \equiv 1$:

$$\tilde{H} = i\sqrt{\frac{\mu_0}{\varepsilon_0}}H \tag{S2}$$

Applying the Bloch theorem within the kez coordinate system, the normalized magnetic and electric fields are expanded along the z axis as follows:

$$\tilde{H} = e^{ik_{\parallel}\cdot r}\sum_{G}\tilde{h}_G e^{i(k_z+G)\cdot z} \tag{S3}$$

$$E = e^{ik_{\parallel}\cdot r}\sum_{G} e_G e^{i(k_z+G)\cdot z} \tag{S4}$$

where $k_{\parallel}$ and k_z represent the in-plane and out-of-plane Bloch wavevectors, respectively. $G = n2\pi/a_z$ is the reciprocal lattice vector for an integer n, and $\tilde{h}_G$ and e_G are the normalized expansion coefficients.

The fundamental Maxwell's equations governing the structural unit are:

$$\nabla \times \tilde{H} = k_0 \varepsilon E \tag{S5}$$

$$\nabla \times E = k_0 \tilde{H} \tag{S6}$$

where k_0 is the wavevector of the excitation light in a vacuum and ε is the material permittivity tensor.

To accurately model the twisted geometry, the permittivity tensors must be transformed from the xyz system to the kez system. For the bottom α-MoO_3 layer, the relationship is given by:

$$[\varepsilon_{kez,bottom}] = F_{bottom}(\varphi)[\varepsilon_{xyz,bottom}]F^{-1}_{bottom}(\varphi) \tag{S7}$$

For the top α-MoO_3 layer, accounting for the twist angle θ, the relationship is:

$$[\varepsilon_{kez,top}] = F_{top}(\varphi-\theta)[\varepsilon_{xyz,top}]F^{-1}_{top}(\varphi-\theta) \tag{S8}$$

where F_{bottom} and F_{top} represent the respective spatial rotation matrices.

By substituting the Bloch expansions into Maxwell's equations within the kez coordinate system, the problem is recast into a generalized eigenvalue matrix equation:

$$\begin{bmatrix} \mathbf{O} & \vdots & \mathbf{I} \\ & \cdots & \\ & \mathbf{P} & \end{bmatrix} \begin{bmatrix} \mathbf{E}_e \\ \mathbf{E}_z \\ \widetilde{\mathbf{k}}_k \mathbf{E}_e \\ \widetilde{\mathbf{k}}_k \mathbf{E}_z \end{bmatrix} = \widetilde{\mathbf{k}}_k \begin{bmatrix} \mathbf{E}_e \\ \mathbf{E}_z \\ \widetilde{\mathbf{k}}_k \mathbf{E}_e \\ \widetilde{\mathbf{k}}_k \mathbf{E}_z \end{bmatrix} \tag{S9}$$

where $\mathbf{O}$ and $\mathbf{I}$ are the zero and identity matrix, respectively. $\widetilde{\mathbf{k}_k} = \mathbf{k}_k/\mathbf{k}_0$ is eigenvalue, and the order of eigenfunction $\begin{bmatrix} \mathbf{E}_e \\ \mathbf{E}_z \\ \widetilde{\mathbf{k}_k}\mathbf{E}_e \\ \widetilde{\mathbf{k}_k}\mathbf{E}_z \end{bmatrix}$ is 4N. N is the number of the set $\{G\}$, and

$$\mathbf{P} = \mathbf{M}^{-1}\left(\mathbf{B_1}\mathbf{S}\left(\mathbf{Q} \vdots \mathbf{D}\right) + \left(\mathbf{B_2} \vdots \mathbf{C_1}\mathbf{S}^{-1}\mathbf{Q}\right)\right) \tag{S10}$$

$$\begin{cases} \mathbf{M} = \left(\mathbf{I} - \mathbf{C_1}\mathbf{S}^{-1}\mathbf{D}\right)^{-1} \\ \mathbf{C_1} = \begin{pmatrix} \widetilde{(\mathbf{k}_z + \mathbf{G})} \\ \widetilde{\mathbf{k}_e} \end{pmatrix} \\ \mathbf{D} = \begin{pmatrix} \widetilde{\mathbf{k}_e} & \widetilde{(\mathbf{k}_z + \mathbf{G})} \end{pmatrix} \\ \mathbf{S} = \widetilde{(\mathbf{k}_z + \mathbf{G})}\widetilde{(\mathbf{k}_z + \mathbf{G})} + \widetilde{\mathbf{k}_e}\widetilde{\mathbf{k}_e} - [\varepsilon_{kk}] \\ \mathbf{Q} = \begin{pmatrix} [\varepsilon_{ke}] & \mathbf{O} \end{pmatrix} \\ \mathbf{B_1} = \begin{pmatrix} \mathbf{O} \\ [\varepsilon_{ek}] \end{pmatrix} \\ \mathbf{B_2} = \begin{pmatrix} \widetilde{\mathbf{k}_e}\widetilde{(\mathbf{k}_z + \mathbf{G})} & \left[\varepsilon_z^{-1}\right]^{-1} - \widetilde{\mathbf{k}_e}\widetilde{\mathbf{k}_e} \\ [\varepsilon_{ee}] - \widetilde{(\mathbf{k}_z + \mathbf{G})}\widetilde{(\mathbf{k}_z + \mathbf{G})} & \widetilde{(\mathbf{k}_z + \mathbf{G})}\widetilde{\mathbf{k}_e} \end{pmatrix} \end{cases} \tag{S11}$$

To improve the convergence of the numerical calculation, the inverse rule is applied to the Toeplitz matrix formulation $[\varepsilon_z^{-1}]^{-1}$.

By directly solving this matrix for its eigenvalues, we can extract the in-plane wavevector magnitude $\mathbf{k}_k$ for any specified propagation direction at a given frequency, allowing us to rigorously derive the IFCs of the twisted bilayer α-MoO_3 configurations. It should be noted that the hole array modifies the effective permittivity of the patterned layer, which is not accounted for in the EPWE model and may therefore introduce a small deviation in the calculated IFCs. To assess the influence of the removed area, we performed additional simulations with reduced hole radii, using the results shown in Figures 2b and 2f as references. As

the hole radius decreases, the dominant diffraction orders in the corresponding momentum-space distributions remain unchanged and consistently match the EPWE-predicted IFCs (Figure S7). These results indicate that the perturbation introduced by the removed area is sufficiently small that it does not cause a substantial displacement of the EPWE-predicted IFCs or lead to misidentification of the relevant diffraction orders.

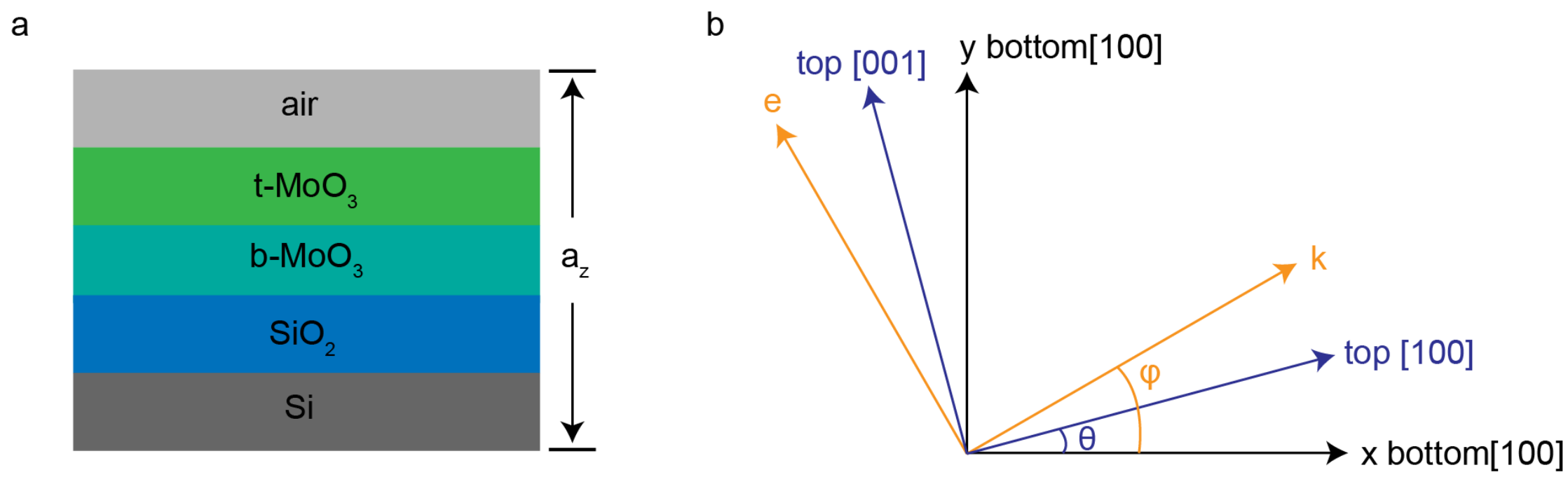

Figure S6. Schematic diagram of the layered structure model used for calculating polariton isofrequency contours using the plane wave expansion method. The stack consists of a silicon (Si) substrate, a silicon dioxide (SiO_2) layer, bottom (b-MoO_3) and top (t-MoO_3) α-MoO_3 layers, and air environment.

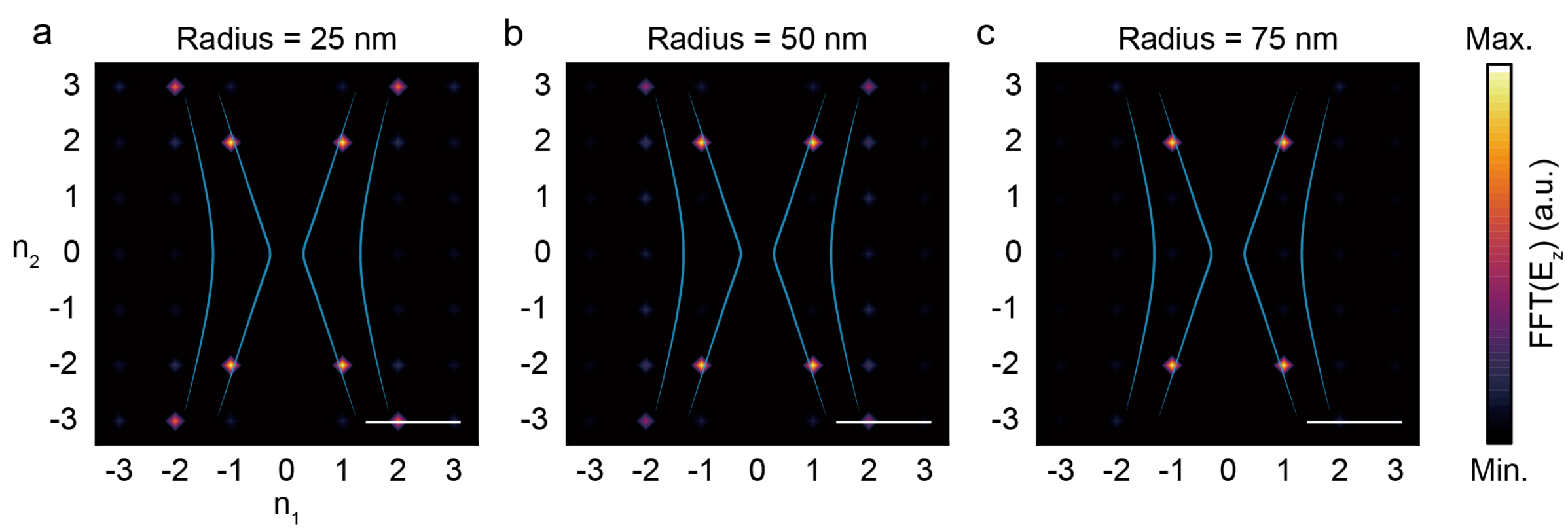

Figure S7. EPWE-calculated IFCs overlaid on FFT amplitude maps derived from simulated real-space electric-field distributions ($Re(E_z)$) above the PoC unit cell for different hole radii.

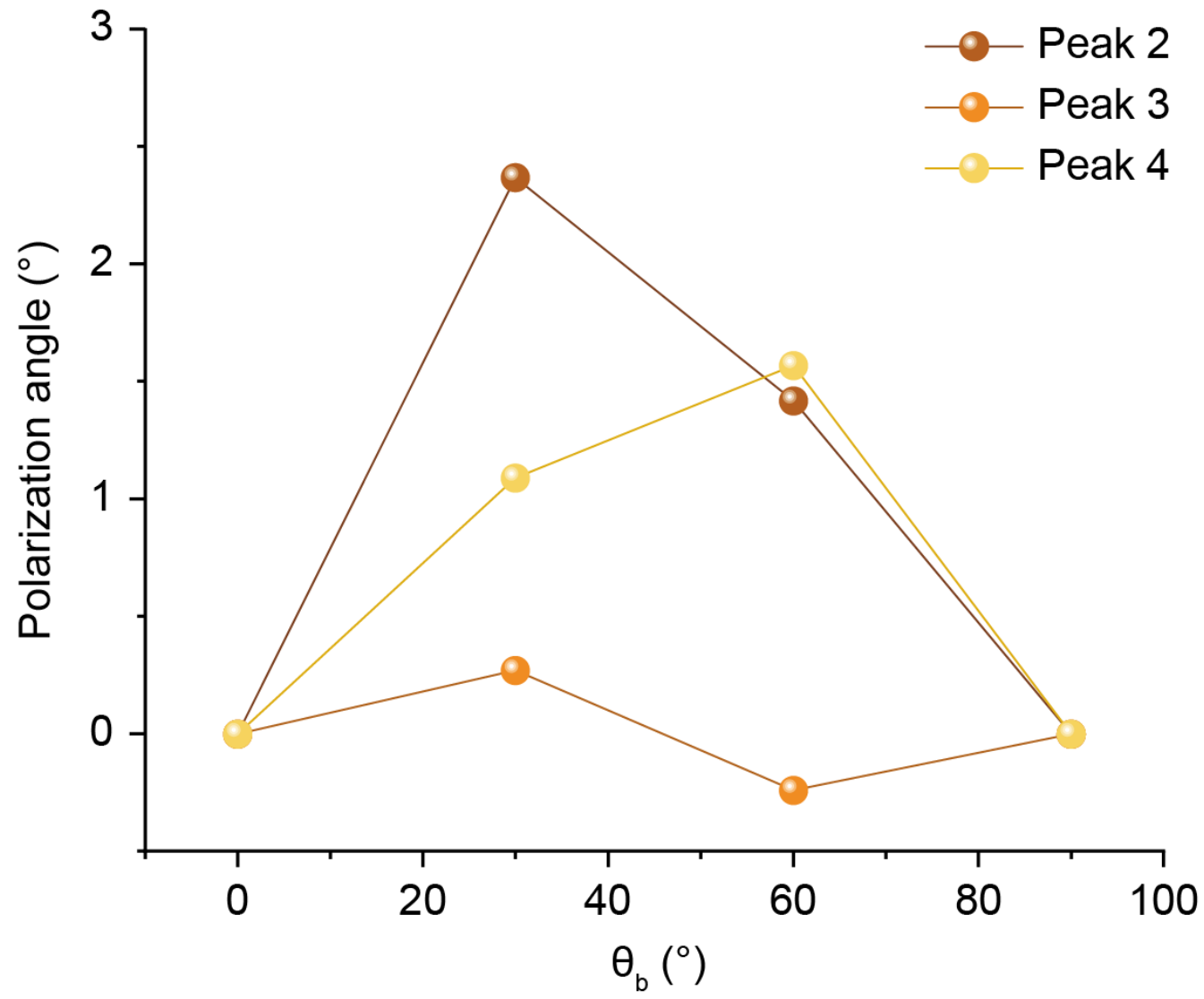

Figure S8. Polarization angles of the maximum absorption axes of the weaker resonances (peaks 2-4) as a function of the bottom-layer twist angle θ_b. The top layer is oriented at $\theta_t = 0°$.

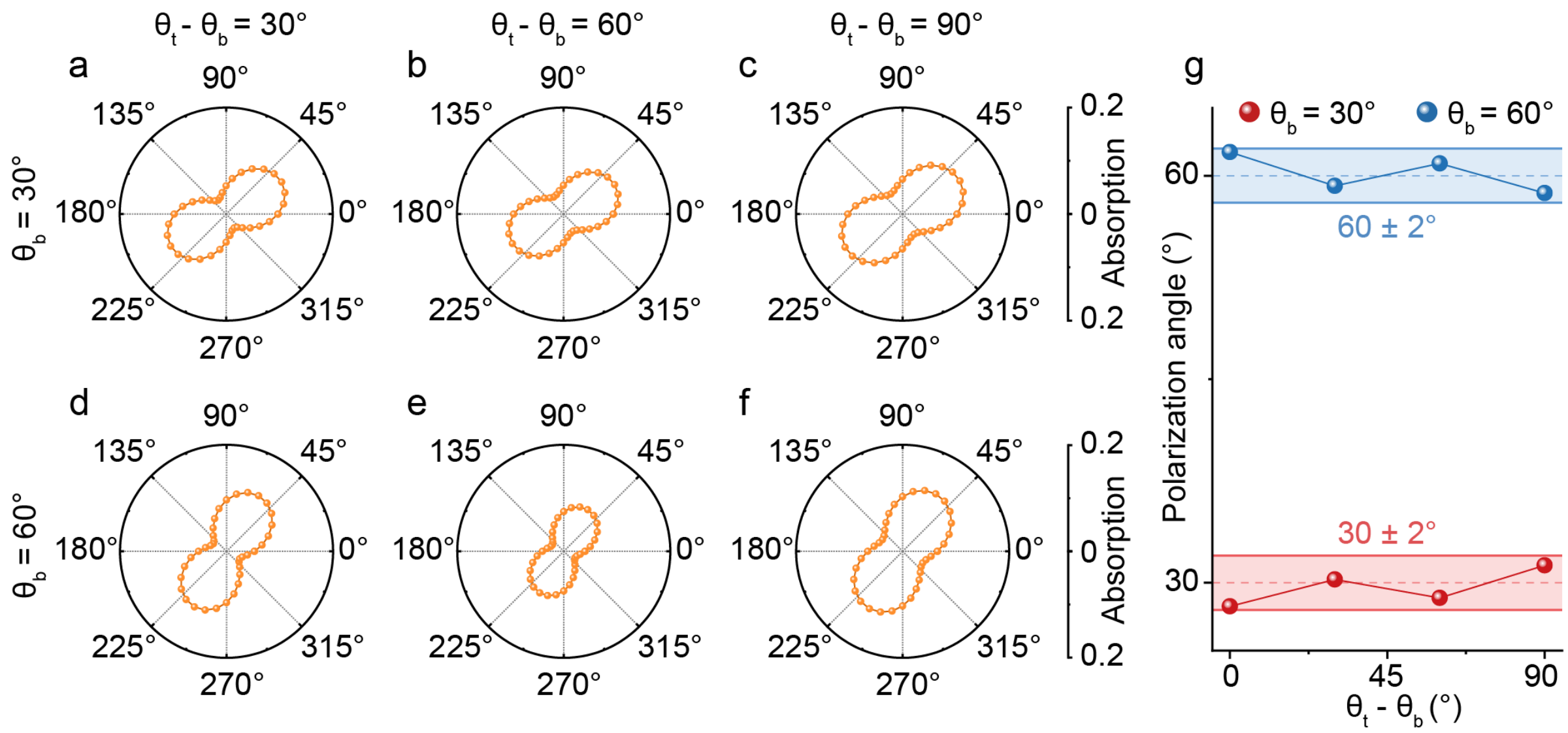

Figure S9. Polarization locking to the [100] crystal axis of the patterned bottom layer. (a–c) Polar plots of the absorption intensity as a function of excitation polarization angle for the primary resonance peak at a fixed bottom-layer twist angle of $\theta_b = 30°$ with relative twist angles of $\theta_t - \theta_b = 30°$, 60°, and 90°. (d–f) Polar plots of the absorption intensity as a function of excitation polarization angle for the primary resonance peak at a fixed bottom-layer twist angle of $\theta_b = 60°$ with relative twist angles of $\theta_t - \theta_b = 30°$, 60°, and 90°. (g) Polarization angles of the maximum absorption axes for the resonance peak as a function of the relative twist angle $\theta_t - \theta_b$ at fixed bottom-layer orientations of $\theta_b = 30°$ (red dots) and 60° (blue dots), where the shaded bands represent the reference ranges of $30 \pm 2°$ and $60 \pm 2°$ respectively.

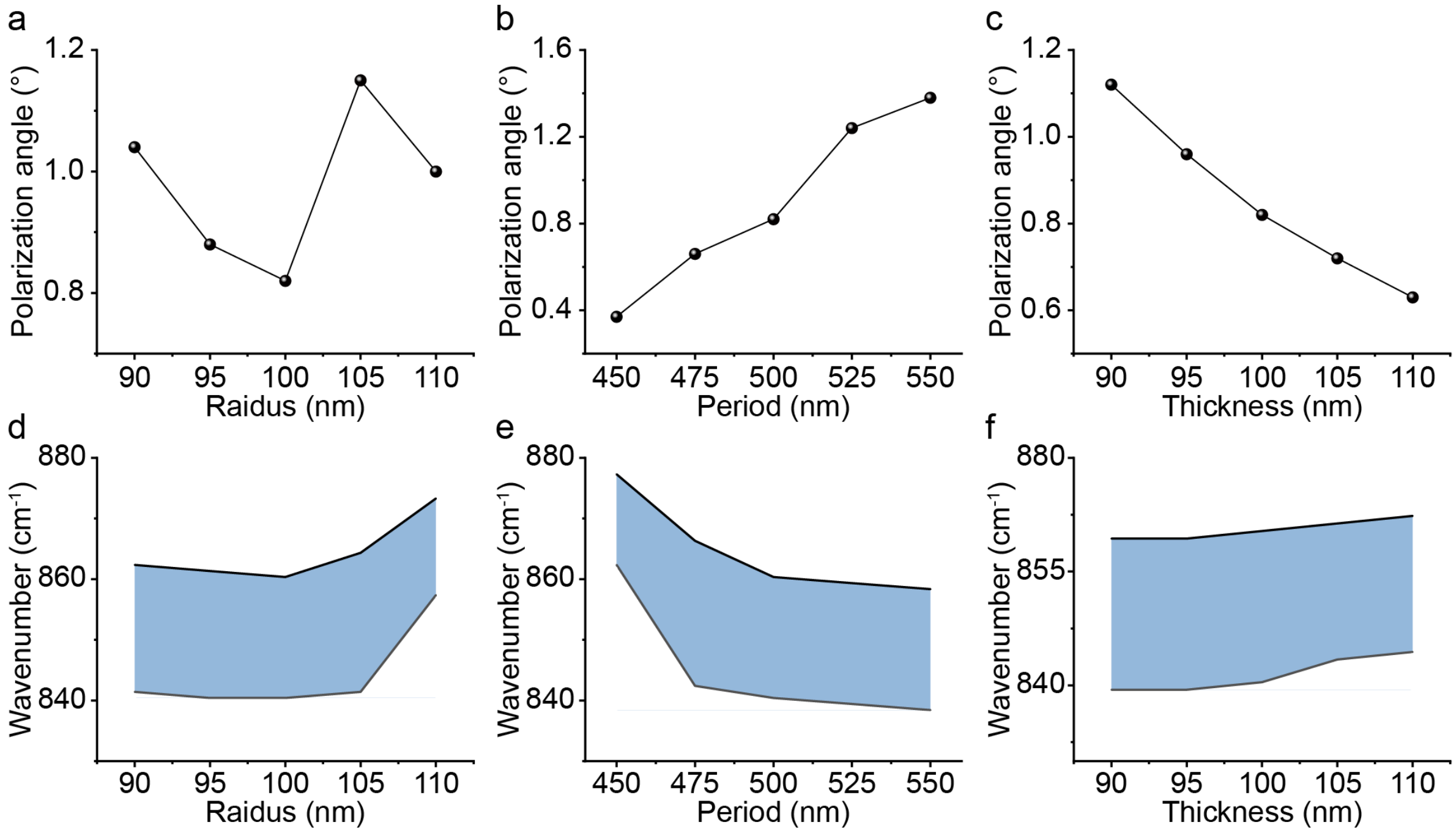

Figure S10. Influence of variations in PoC structural parameters on polarization locking and spectral tuning range. The top and bottom layers are oriented at θ_t = 0° and θ_b = 30°, respectively. (a–c) Polarization angles of the maximum absorption axes of the primary resonance peak as a function of the hole radius, lattice period, and patterned-layer thickness, respectively. (d–f) Corresponding spectral tuning ranges of the primary resonance peak under the same structural-parameter variations.

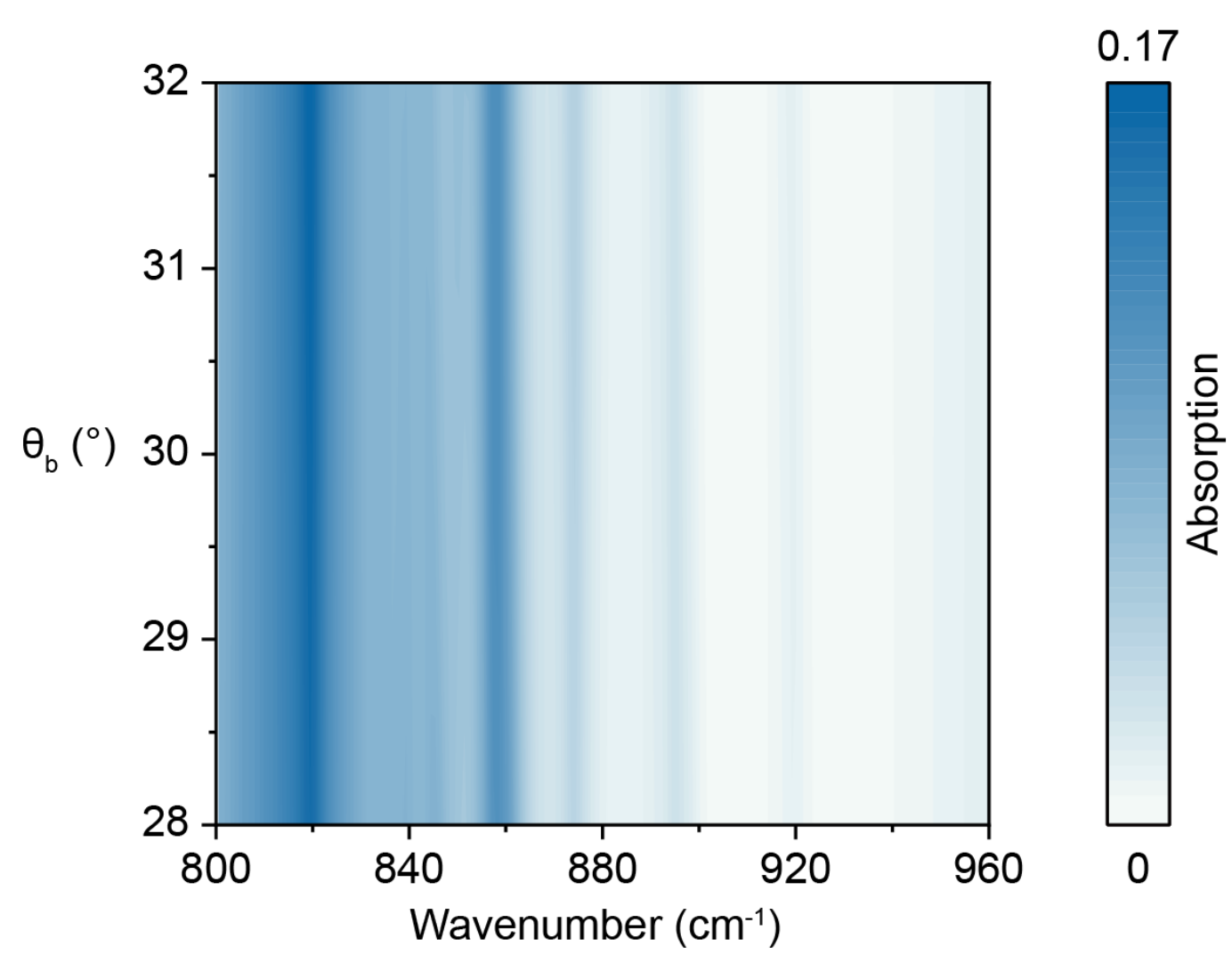

Figure S11. Influence of twist-angle deviations on the spectral response. Simulated absorption spectra evaluating the effect of ±2° angular deviations in θ_b from 28° to 32° relative to the reference state with θ_t = 0° and θ_b = 30°.

References

(1) Chen, M.; Lin, X.; Dinh, T. H.; Zheng, Z.; Shen, J.; Ma, Q.; Chen, H.; Jarillo-Herrero, P.; Dai, S. Configurable phonon polaritons in twisted α-MoO_3. *Nat. Mater.* **2020**, *19*, 1307–1311.

(2) Zhou, L.; Ni, X.; Wang, Z.; Renzi, E. M.; Xu, J.; Zhou, Z.; Yin, Y.; Yin, Y.; Song, R.; Zhao, Z.; others Engineering shear polaritons in 2D twisted heterostructures. *Nat. Commun.* **2025**, *16*, 2953.

(3) Hsue, Y.-C.; Freeman, A. J.; Gu, B.-Y. Extended plane-wave expansion method in three-dimensional anisotropic photonic crystals. *Phys. Rev. B* **2005**, *72*, 195118.
